# Fluorescent Molecular Logic Gates and Pourbaix Sensors in Polyacrylamide Hydrogels

**DOI:** 10.3390/molecules27185939

**Published:** 2022-09-13

**Authors:** Glenn J. Scerri, Melchior Caruana, Nicola’ Agius, Godfrey Agius, Thomas J. Farrugia, Jake C. Spiteri, Alex D. Johnson, David C. Magri

**Affiliations:** Department of Chemistry, Faculty of Science, University of Malta, MSD 2080 Msida, Malta

**Keywords:** hydrogel, polyacrylamide, fluorescence, photoinduced electron transfer, internal charge transfer, molecular logic gate

## Abstract

Polyacrylamide hydrogels formed by free radical polymerisation were formed by entrapping anthracene and 4-amino-1,8-naphthalimide fluorescent logic gates based on photoinduced electron transfer (PET) and/or internal charge transfer (ICT). The non-covalent immobilisation of the molecules in the hydrogels resulted in semi-solid YES, NOT, and AND logic gates. Two molecular AND gates, examples of Pourbaix sensors, were tested in acidic aqueous methanol with ammonium persulfate, a strong oxidant, and displayed greater fluorescence quantum yields than previously reported. The logic hydrogels were exposed to aqueous solutions with chemical inputs, and the fluorescence output response was viewed under 365 nm UV light. All of the molecular logic gates diffuse out of the hydrogels to some extent when placed in solution, particularly those with secondary basic amines. The study exemplifies an effort of taking molecular logic gates from homogeneous solutions into the realm of solid-solution environments. We demonstrate the use of Pourbaix sensors as pE-pH indicators for monitoring oxidative and acidic conditions, notably for excess ammonium persulfate, a reagent used in the polymerisation of SDS-polyacrylamide gels.

## 1. Introduction

Hydrogels are three-dimensional macromolecular polymers consisting of a network of cross-linked polymer chains with large amounts of water tightly held within the polymer structure [1]. The polymer network is permeable to water and allows for the diffusion of gases and liquids. The polymeric structure has a high porosity and soft consistency analogous to the texture and flexibility of biological tissues [2]. These properties make hydrogels advantageous for biomedical products such as contact lenses, biosensors, and tissue engineering [3]. The first hydrogels were developed by Wichterle and Lím over half a century ago [4]. Interest in these remarkable materials remains strong for the development of drug delivery devices, chromatographic packing, and electrophoresis gels [5].

**Figure 1:**
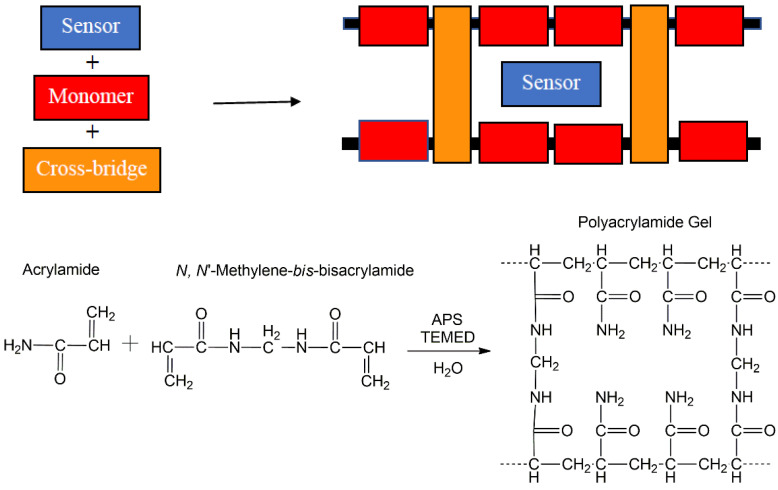
The concept of a sensor (chemosensor, logic gate) encapsulated within a pore of a cross-linked water-compatible polymer matrix (**top**). The polymer is synthesised from acrylamide (monomer) and *N*,*N*′-methylene-*bis*-acrylamide (cross-bridge) using APS and TEMED to form the polyacrylamide hydrogel (**bottom**).

Polyacrylamide hydrogels are used routinely in sodium dodecyl sulphate (SDS)-polyacrylamide gel electrophoresis (PAGE) for separating proteins and DNA [6]. The hydrogels are formed by free radical polymerisation of acrylamide (monomer) and *N*,*N′*-methylene-*bis*-acrylamide (cross-linker) by vinyl addition typically using TEMED (*N*,*N*,*N′*,*N′*-tetramethylethylenediamine) as the catalyst and ammonium persulfate (APS) as the initiator in water. During the polymerisation process, the persulfate generates free radicals that react with the monomer and the cross-linker forming a porous material with cavities able to encapsulate sensor molecules (Figure 1).

In addition to acting as an oxidant, ammonium persulfate acts as a buffer between pH 8 and 10 (p*K*_a_ (NH_4_^+^) = 9.2). At lower pH, TEMED (p*K*_a1_ = 8.97) becomes protonated, which slows the initiation process as the unprotonated form is required for initiating polymerisation. A practical concern during SDS-PAGE studies is that excess persulfate may cause the oxidation of proteins and nucleic acids, especially sulfhydryl-containing compounds [6]. Hence, a fluorescent method for monitoring excess persulfate could be useful to biochemists and protein scientists. An alternative procedure uses riboflavin (vitamin B2) as the free radical source after activation with light and oxygen to initiate polymerisation. A major advantage of this latter approach, known as photochemical polymerisation, is that riboflavin is active at a lower concentration range of 10 ug mL^−1^. However, a disadvantage of this method is that it takes much longer for complete polymerisation to occur—up to 8 h versus 60 min by the TEMED/APS combination.

Fluorescent chemosensors and logic gates have been featured in hydrogels for a variety of applications [7,8,9,10,11,12]. McCoy developed hydrogels with luminescent pH-modulated (YES logic gate) Eu(III)-based quinoline cyclens [13]. A fluorogenic polyacrylamide sensor embedded with 6,7-dihydrocoumarin was used as a *turn-off* sensor (NOT logic gate) for the nerve agent diethylchlorophosphate [14]. Borisov demonstrated photoinduced electron transfer (PET)-based optical sensors for K^+^ [15,16], Na^+^ [17], CO_2_ [18], and H^+^ [18,19] in polyurethane hydrogels. Thapa reported PET-based crown ethers for sensing Ba^2+^ in polyacrylamide [20]. Gunnlaugsson described p(HEMA-co-MMA) lanthanide-based hydrogel as a H^+^, F^−^-driven logic circuit with IMPLICATION, NOR, and TRANSFER functions with dual fluorescence and phosphorescence modes [21]. Nandi communicated a riboflavin-methyl cellulose hydrogel as a semi-solid H^+^,T-driven AND logic gate (T = temperature) [22]. We hypothesise that molecules able to perform logic-based computations [23] could be useful tools for developing smart materials for a variety of applications.

**Figure 2:**
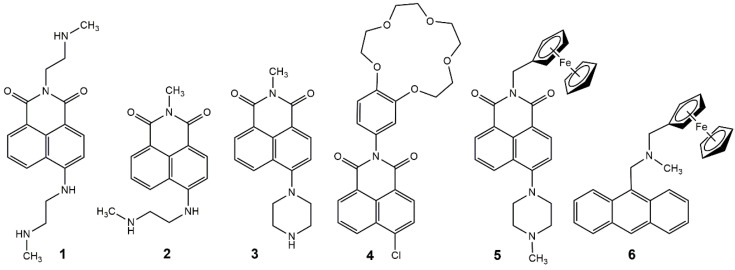
The molecules studied in cross-linked polyacrylamide hydrogels.

In this study, we embed fluorescent molecules **1-6** (Figure 2) based on photoinduced electron transfer and/or internal charge transfer in polyacrylamide hydrogels. The hydrogels embedded with **1–6** function as YES [24], NOT [25], and AND [26,27] logic gates. Molecules **1–3** are single-input gates with a H^+^ input, and **4** is a single-input gate with a Na^+^ input. Molecules **5** and **6** are demonstrated as H^+^ and S_2_O_8_^2^^−^ double-input AND gates. The latter are examples of Pourbaix sensors, which are responsive to pH and potential (pE) [28]. In past studies, we used Fe^3+^ as the oxidant, for example, with ferrocenyl-pyrazoline INHIBIT logic gates [29]. Recently, we substituted APS for Fe^3+^ as the oxidant and observed a significant fluorescence enhancement, a 10-fold increase, from a Φ_f_ from 1.8% to 19.2% [30]. Herein, we share new results on Pourbaix sensors **5** and **6** with APS as the oxidant in the solution. Furthermore, we propose and explore the possibilities of using Pourbaix sensors as fluorescent tools for monitoring ammonium persulfate (NH_4_)_2_S_2_O_8_ in polyacrylamide hydrogels.

## 2. Results and Discussion

### 2.1. Homogeneous Solution Studies

Molecules **1–3** are yellow-emitting fluorescent pH indicators [24]. The compounds function by an excited internal charge transfer (ICT) state with a dipole moment of 10 D. In the UV–vis spectra, isosbestic points are evident at 441 nm, 440 nm, and 393 nm. The molecules emit at ca. 540 nm with fluorescent quantum yields (Φ_f_) of 0.47, 0.58, and 0.50 in water. The excited state p*K*_a_s are 9.3, 9.0, and 9.0, so the compounds are protonated in water. Of the group, **3** is the most sensitive, with a 500-fold enhancement, while **1** and **2** have more modest enhancements of eight and thirteen-fold. These compounds were selected because of their solvatochromic properties as they emit different colours depending on the solvent polarity: blue in hexane, green in diethyl ether, and yellow in water [24].

Molecule **4** is a blue-emitting Na^+^ indicator [25]. In 1:1 (*v*/*v*) methanol/water, there is a maximum of 345 nm in the UV–vis absorbance spectrum. No isosbestic point is observed on titration with Na^+^, which is ideal for a photo-induced electron transfer sensor. Initially, the molecule is weakly fluorescent with a λ_Flu_ of 406 nm and Φ_F_ of 0.02. Upon titration of Na^+^, binding occurs at the benzo-crown ether with a p*β*_Na+_ of 0.81 and the emission of the Na^+^-bound molecule becomes diminished and undetected by the naked eye with a Φ_F_ of 0.0029. Consequently, **4** functions as an *on-off* switch or a NOT logic gate. For sensing purposes, *off-on* switches or YES logic gates, such as Heagy’s 4-sulfon-1,8-naphthalic anhydride analogue tend to be more appreciated [31]. Bi-functional fluorescent *turn-on* probes for hydrophobicity and Na^+^, such as Toyo’oka’s 4-*N*-(4′-aminomethylbenzo-15-crown-5)-7-nitro-2,1,3-benzoxadiazole, would complement this study nicely [32].

Molecules **5** and **6** are green and blue-emitting Pourbaix sensors with λ_Flu_ at 526 nm and 395 nm [25,26]. The excited state p*K*_a_s of **5** and **6** are 6.6 (1:1 MeOH/water) and 7.8 (water) [26]. The Φ_f_ in acidic solutions are 0.086 and 0.018 in methanol/water and methanol, respectively, based on Fe^3+^ as the oxidant. A goal of this study was to evaluate the AND logic function of our Pourbaix sensors using APS as the oxidant. Ammonium persulfate is a much stronger oxidant than Fe^3+^ with an oxidation potential of *E*° = +2.1 V versus *E*° = +0.77 V. Advantageously, APS readily dissolves in water across the pH scale and is transparent and colourless in the UV–visible region, unlike Fe^3+^, which has a tendency to hydrolyse to insoluble Fe(OH)_3_ above pH 4 and absorbs to some extent in the UV region. APS also transfers two-mole equivalents of electrons and the oxidation product is sulphate, a relatively inert anion. A summary of the photophysical parameters of **1–6** is tabulated in Table 1.

Table 2 summarises the truth tables for **5** and **6** according to the possible permutations of acid and APS chemical inputs. In the case of **5**, we observe an impressive 65-fold fluorescence enhancement between the most fluorescent state (1,1) and the second highest (1,0). A substantially brighter emission is observed using APS rather than Fe^3+^ as the oxidant in 1:1 (*v*/*v*) methanol/water with a Φ_f_ of 39.1%. With Fe^3+^ as the oxidant, the Φ_f_ was limited to 8.6% (Table 1). This difference is due to the strength of APS as an oxidant and the fact that it is colourless and transparent in solutions at millimolar concentrations. The concentration of Fe^3+^ was limited to micromolar concentrations due to absorption effects at the excitation wavelength [27].

**Figure 3:**
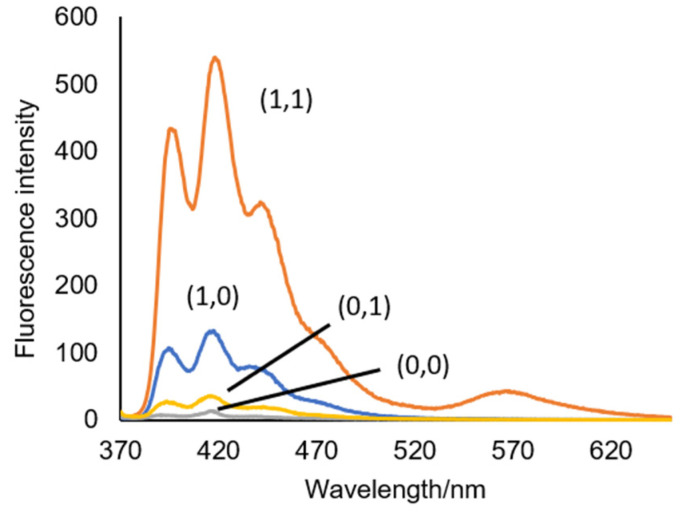
Emission spectra of **6** in 1:9 (*v*/*v*) MeOH/H_2_O excited at λ_ex_ = 368 nm. The numbers in parentheses are the binary input conditions, as given in Table 2.

With **6**, we first tested the AND logic behaviour in methanol using methanesulfonic acid and APS as the oxidant. The UV–visible absorbance spectra have characteristic anthracene peaks at 364 nm and 385 nm. The addition of acid causes a 4–5 nm red-shift to 369 nm and 389 nm and isosbestic points at 361 nm, 368 nm, 381 nm, and 387 nm. These observations are consistent with our published results in methanol using Fe^3+^ as the oxidant [26]. We wish to highlight the enhanced Φ_f_ = 0.0834 and the 8-fold fluorescence enhancement with APS compared to a Φ_f_ = 0.018 and a 4.5-fold enhancement with Fe^3+^. As the hydrogel environment is hydrophilic, we felt obliged to also examine **6** under majority aqueous conditions. We prepared a 1:9 (*v*/*v*) MeOH/H_2_O solution of **6**. In the UV–vis spectra, we noticed that the anthracene peaks were not so resolved. An emission scan of **6** excited at **λ**_ex_ = 368 nm in the presence of 10**^−^**^4^ M acid and 5.0 mM APS gave the expected peak profile at 417 nm and 424 nm. However, we also observed a broad, structureless band between 530–650 nm with a **λ**_max_ of 568 nm (Figure 3).

These observations are consistent with anthracene monomer and excimer emission. Ground-state aggregates were invoked in the study of anthracene *ortho*-aminomethylphenylboronic and anthraceneaminomethylphenyl sensors in 1:2 (*v*/*v*) methanol/water to explain the observation of an excimer, which is absent in methanol [33]. We thus proceeded to increase the proportion of methanol to give a 1:4 (*v*/*v*) MeOH/H_2_O solution and observed the peak at 417 nm increase at the expense of a decrease in the broad band at 568 nm. Accordingly, the monomer Φ_f_ in the presence of acid and APS in 10% MeOH is 0.0533 and in 20% MeOH is 0.0834 (Table 2). Dilution to 30% MeOH results in the monomer emission decreasing slightly and the tail portion of the spectrum beyond 540 nm remaining nearly identical. Examined under a 365 nm UV lamp, the 30% MeOH solution colour of **6** with H^+^ and APS is pinkish rather than blue [25], suggesting the remnants of aggregates.

### 2.2. Hydrogel Studies

**Figure 4:**
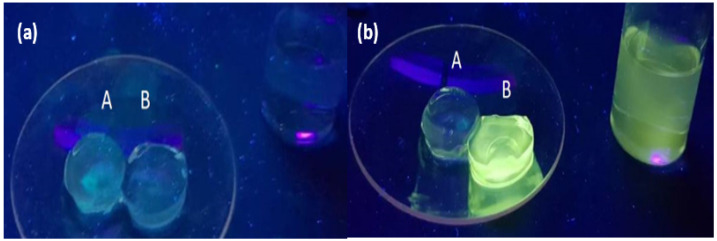
(**a**) Hydrogel pellets A and B (1.8 cm diameter). Sample A is a blank polyacrylamide control (PASS 0 logic gate). Sample B is embedded with **3** and exposed to a 0.1 M NaOH solution. (**b**) Sample B after exposure to 0.1 M HCl (beaker, right side). A yellow fluorescence is observed due to protonation of the amine receptor, preventing PET. The acid solution is also fluorescent due to diffusion of **3** from the hydrogel. The samples are irradiated with 365 nm UV light.

Molecules **1–6** were embedded in the hydrogels in situ by mixing sensor solutions with the monomers prior to polymerisation. The polyacrylamide gels were formed by polymerisation of acrylamide and *N,N′*-methylene-*bis*-acrylamide by a free radical reaction initiated by ammonium persulfate via vinyl addition of acrylamide at ambient temperature in aqueous conditions. Details are available in the Section 3. The percent weight of the cross-linker was 0.08%, providing visually transparent hydrogels [1]. In all studies with the polyacrylamide hydrogels, the fluorescence of **1–3** was yellow, confirming that the environment within the hydrogel is indeed hydrophilic (Figure 4).

**Figure 5:**
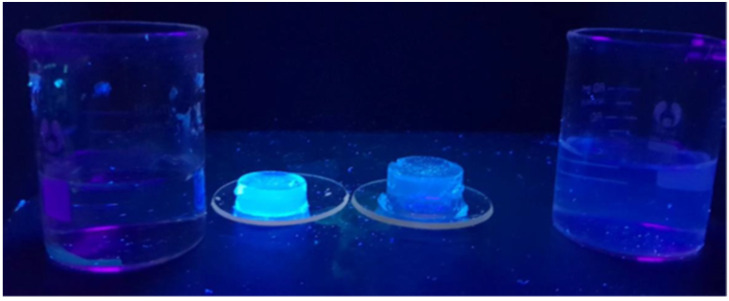
Two polyacrylamide pellets with 60 μM **4** irradiated with 365 nm light after 1 day of exposure to aqueous solutions. (Left) pellet of **4** after exposure to deionised water and (right) after exposure to aqueous 1 M NaCl solution. Na^+^-driven *on–off* (NOT) logic is observed. The supernatant in the beaker (right) emits blue emission due to leaching of **4** and incomplete saturation of the benzocrown receptor.

**Figure 6:**
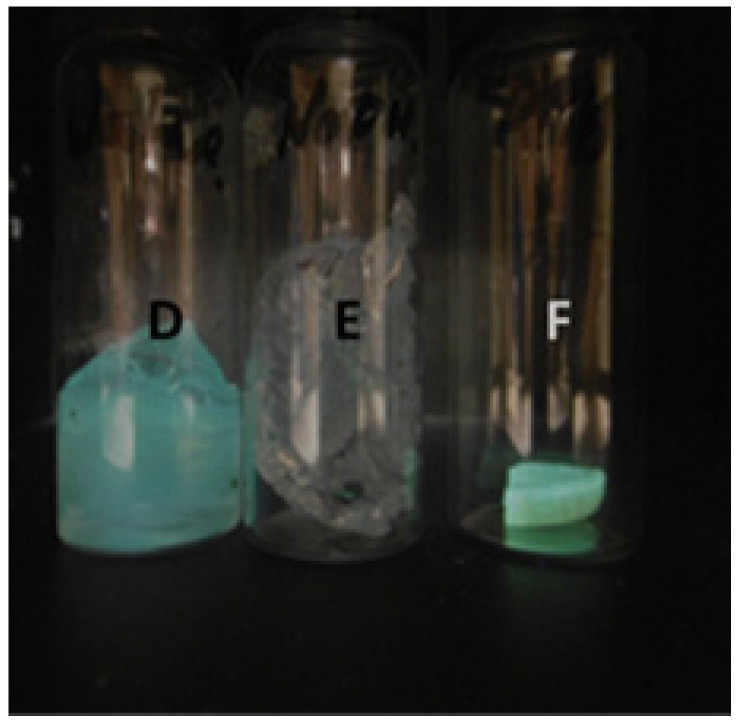
Vials D-F irradiated with 365 nm UV light in a dark cabinet. Vial D contains a hydrated polyacrylamide hydrogel embedded with **5** in the presence of APS. Vial E contains a hydrated hydrogel with **5**, APS and 0.5 M NaOH solution, which turns off the fluorescence. Vial F contains a dried fragment of solid polyacrylamide with **5** emitting a green fluorescence.

In Figure 5, a distinctive blue emission is emitted from the polyacrylamide hydrogel (left pellet). During the polymerisation of acrylamide, APS is consumed in the reaction. However, (NH_4_)_2_S_2_O_8_ in the polymeric matrix is also detected by Pourbaix sensors **5** and **6**. Hence, we observed that on hydrogel formation, **5** is in the *on* state (Figure 6). Pourbaix sensor **5** senses for the oxidising agent APS and acidity by providing a green fluorescence output. Ideally, the fluorescence would be sustained until all the APS is consumed and no more oxidant remains to oxidise the ferrocene moiety to the ferrocenium radical cation. However, if excess APS remains, a green emission is still observed. Hence, in principle, the Pourbaix sensors act as reaction indicators.

Steady-state and time-resolved fluorescence studies with 4-aminophthalimide indicate that there are multiple microenvironments inside polyacrylamide hydrogels [34]. This arises from the inhomogeneity of the gel matrix and a distribution of pore sizes. Numerous studies indicate that the mean pore size is dependent on the amount of cross-linker, which decreases with increasing monomer concentration [35,36,37]. The approximate diameter of anthracene is 1.1 nm, and the longest axes of the amino-1,8-naphthalimide molecules **1**, **4**, and **5** are estimated to be no longer than 2.2 nm. Hence, the pores sizes are anticipated to be large enough to allow these nanometre-sized molecules to diffuse about and out of the hydrogel. It is therefore not surprising that diffusion of the molecules was detected on UV irradiation of the supernatant solutions resulting from the washed hydrogels and confirmed by running fluorescence spectra on the supernatant.

We attempted to obtain emission spectra of **1–6** within hydrogels prepared in 10 mm Suprasil quartz cuvettes. Although macroscopically transparent, almost no emission was observed from the hydrogels impregnated with the molecules. For example, while an intense fluorescence is observed from **3** in an aqueous solution, the majority of the emission is blocked, embedded in the bulk hydrogel. The transmittance of polyacrylamide hydrogel is dependent on its cross-linker concentration, thickness, and transmittance wavelength [1]. In future work, we will explore using thinner polyacrylamide hydrogel films [14] and fluorescent molecular logic gates emitting at longer wavelengths [16].

## 3. Materials and Methods

### 3.1. Materials

Acrylamide (99%, Sigma-Aldrich, St. Louis, MO, USA), *N,N′*-methylene-*bis*-acrylamide (99%, Sigma-Aldrich), *N,N,N′,N′*-tetramethylethylene diamine (TEMED, Merck Millipore, Burlington, MA, USA), ammonium persulfate (APS ≥ 98%, Sigma-Aldrich), methanol (Carlo Erba, HPLC grade, Cornaredo, Italy), hydrochloric acid (Thermo Fisher Scientific, 37.5%, Waltham, MA, USA), sodium hydroxide pellets (Thermo Fisher Scientific, Analytical Grade), and tetramethylammonium hydroxide (TMAOH, 25% in H_2_O, Sigma-Aldrich) were used as received. Molecules **1–6** were previously synthesised [24,25,26,27].

### 3.2. Hydrogel Synthesis

The polyacrylamide hydrogels were prepared by mixing aqueous aliquots from three 100 mL stock solutions. The first solution was **A** acrylamide (8.0 g, 1.1 mM) and *N,N,N′N′*-methylene-*bis*-acrylamide (0.15 g, 10 mM) in water. The second solution **B** contained (NH_4_)_2_S_2_O_8_ (1.5 g, 66 mM), and the third solution **C** contained TEMED (1.0 g, 86 mM). Another solution **D** was prepared for the fluorescent logic gate (~10 mg, 20 μM, dissolved in water or 1 mL of methanol). The solutions were purged with nitrogen gas to remove oxygen. The four solutions **A–D** were mixed in a ratio of 5:2:1:2. The solution was poured into 5 mL beakers. The polymerised hydrogels were immersed in deionised water overnight to remove unreacted monomers.

### 3.3. Instrumentation

UV–visible absorption spectra were measured with a Jasco V650 spectrophotometer with Spectra Manager Suite^®^ software. The parameters were set to medium response, a 1 nm bandwidth, and a scan speed of 200 nm min^−1^. Spectra were background subtracted for the solvent. Samples were measured in 10 mm Suprasil^®^ cuvettes in a parallel beam set-up. Fluorescence spectra were recorded with a Jasco FP-8300 spectrofluorometer with Spectra Manager Suite^®^ software. The excitation and emission slits were 2.5 nm. The scan rate was 200 cm^−1^. The pH meter was calibrated using HANNA^®^ pH 7.01 and pH 4.01 buffers prior to any readings.

## 4. Conclusions

Six fluorescent molecules were embedded within polyacrylamide hydrogels and examined as semi-solid logic gates. Molecules **1–3** are water-soluble H^+^-driven YES logic gates with a high fluorescence quantum yield. Molecule **4** is a Na^+^-driven NOT logic gate, and **5** and **6** are H^+^, S_2_O_8_^2-^-driven AND logic gates. All of the molecules have a tendency to diffuse from the hydrogels into the bulk solution. To accommodate this issue, we could covalently link the molecules directly to the polymer matrix. The use of APS as an oxidant results in a four-fold increase in the fluorescent output of Pourbaix sensors **5** and **6** compared to previous studies using Fe^3+^ as the oxidant. These latest results consolidate the potential of Pourbaix sensors as promising tools for biological and material science applications.

## Figures and Tables

**Table 1:** Photophysical data **1–6** from reported literature sources in solution [24,25,26,27].

Parameters	1 ^a^	2 ^a^	3 ^a^	4 ^b^	5 ^b^	6 ^c^
λ_Abs_ (pH 4)/nm	434	433	387	345	386	349
λ_Flu_ (pH 4)/nm	540	538	538	406	526	395
Φ_Fmax_	0.47	0.58	0.50	0.02	0.086	0.018
p*K*_a_*, p*β*_H+_^*^	9.3	9.0	9.0	−	6.6	7.8 ^d^

^a^ Water. ^b^ 1:1 (*v*/*v*) methanol/water ^c^ Methanol. ^d^ Taken from a model compound in water. The excited state p*K*_a_ (p*K*_a_^*^) refers explicitly to binding constants in water while in solvent mixtures the designation p*β*_H+_^*^ is used.

**Table 2:** Truth tables for **5** in 1:1 (*v*/*v*) methanol/water and **6** in 1:4 (*v*/*v*) methanol/water with HCl and APS as inputs and emission as the output ^a^.

Input_1_ ^b^ (H^+^)	Input_2_ ^c^ (S_2_O_8_^2−^)	Output 5 (Φ_f_)	Output 6 (Φ_f_)
0	0	0 (0.001)	0 (0.0002)
1	0	0 (0.006)	0 (0.0101)
0	1	0 (0.001)	0 (0.0011)
1	1	1 (0.391)	1 (0.0834)

^a^ 2 μM **5** and 13 μM **6**, ^b^ For **5**: high H^+^ level 10^−4^ M HCl. Low H^+^ level at 10^−10^ M adjusted with 0.50 M NaOH. For **6**: high H^+^ level 10^−3^ M CH_3_SO_3_H. Low H^+^ level at 10^−10^ M adjusted with TMAOH. ^c^ High and low (NH_4_)_2_S_2_O_8_ levels 5.0 mM and 0 mM, respectively.
